# Screening White-Rot Fungi and Characterizing Lignocellulose Degradation by *Ganoderma* sp. from Chinese Distillers’ Grains

**DOI:** 10.3390/jof12080574

**Published:** 2026-08-03

**Authors:** Yanling Hu, Lingzhen Zhou, Ya Wang, Yuyin Cai, Xiaofeng Guan, Feiyun Yang, Zuohua Liu, Chengling Bao

**Affiliations:** 1 Chongqing Academy of Animal Science, Chongqing 402460, China; yanlh2022@163.com (Y.H.); 15287311358@163.com (L.Z.); wangya920708@163.com (Y.W.); caiyuyin1995@163.com (Y.C.);; 2 National Center of Technology Innovation for Pigs, Chongqing 402460, China; 3 College of Animal Science and Technology, Southwest University, Chongqing 402460, China

**Keywords:** lignocellulose, white-rot fungi, *Ganoderma* sp., Chinese distillers’ grains, laccase

## Abstract

Chinese distillers’ grains (CDGs) are a lignocellulose-rich agricultural byproduct with considerable bioconversion potential; however, their recalcitrant structure imposes a major bottleneck for efficient valorization. In this study, six white-rot fungal strains and two *Trichoderma* strains were isolated from wild macrofungi, among which *Ganoderma* sp. 2G1-6 exhibited the highest laccase activity, reaching 11.29 U/mL. Whole-genome sequencing revealed a 49.15 Mb genome containing 12,437 predicted proteins, including a substantial repertoire of 196 glycoside hydrolases and 113 auxiliary activity enzymes implicated in lignocellulose degradation. Solid-state fermentation of CDGs with *Ganoderma* sp. 2G1-6 produced high enzyme activities, including endoglucanase (13.3 ± 4.6 U/g), xylanase (538.8 ± 33.0 U/g), β-glucosidase (26,572 ± 2996 U/g), and laccase (231.2 ± 16.7 U/g). Concomitantly, the nutritional profile of CDGs was markedly improved after 30 days, with crude protein increasing by 28.51%, acid-soluble protein by 33.33%, and soluble dietary fiber by 159%. Complementary structural analyses (FTIR, SEM, XRD, and TGA) confirmed progressive disruption of the lignocellulosic matrix, selective delignification, and enhanced cellulose exposure. These findings establish *Ganoderma* sp. 2G1-6 as a promising microbial chassis for CDGs valorization and provide preliminary functional profiles of *Ganoderma* species for lignocellulose bioconversion.

## 1. Introduction

Global economic and social development requires large energy inputs, intensifying pressure on energy supplies [1]. Lignocellulosic biomass, owing to its abundance and carbon-neutral nature, is recognized as a pivotal feedstock for the production of biofuels and value-added bioproducts [2]. Chinese distillers’ grains (CDGs), a biological waste generated from the traditional baijiu fermentation industry, are rich in lignocellulose. With an annual output exceeding 20 million tons [3], CDGs pose significant environmental management challenges, yet they also offer a substantial resource base for use as a feedstock in bioproduct and biofuel manufacturing.

CDGs hold notable potential for biomass energy production (e.g., fuel ethanol and biogas) and as a feed ingredient, due to their substantial availability [4]. Nutritionally, they are rich in protein (15.21–16.75%) and contain residual fats, starch, minerals, and bioactive phytochemicals such as phenolic acids and flavonoids [5,6,7,8,9]. However, the efficient utilization of these nutrients is severely compromised by the high rice husk content (40–50% of air-dried matter). This results in a recalcitrant lignocellulosic complex, wherein cellulose (19.34%) and hemicellulose (7.89%) are encased within a protective lignin matrix (10.68%) [10]. The chemical cross-linking between lignin and hemicellulose forms a physical barrier that impedes enzymatic access and reduces microbial digestibility [11].

Consequently, current disposal methods are either economically prohibitive or environmentally unsustainable [12]. The development of efficient pretreatment strategies to deconstruct the lignocellulosic architecture of CDGs is therefore essential for enhancing their value, particularly in the feed industry. While physical and chemical pretreatments exist, they are often associated with high operational costs or the generation of inhibitory by-products [13]. Biological pretreatment via microbial fermentation operates under mild conditions and represents an environmentally benign alternative. However, conventional industrial microorganisms—including *Aspergillus* spp., yeasts, and lactic acid bacteria—typically exhibit limited lignocellulolytic capacity, underscoring the need to identify more potent and specialized fungal degraders.

The degradation of lignin, as the primary recalcitrant component, constitutes the rate-limiting step in lignocellulose bioconversion. Unlike most microorganisms that target only cellulose and hemicellulose, white-rot fungi (WRF) possess a unique, non-specific oxidative ligninolytic system capable of extensive lignin mineralization [14]. This enzymatic machinery, often triggered by nutrient limitation, acts synergistically to dismantle all components of the lignocellulosic matrix [15]. Applied research has corroborated this potential: solid-state fermentation (SSF) employing WRF has been shown to effectively reduce lignin content and improve protein digestibility in agricultural residues such as corn stover [16] and white tea residue [17].

Despite these advances, the potential of WRF for degrading CDGs remains largely unexplored. CDGs differ considerably from typical agricultural straws, particularly owing to their high content of silica-rich rice husk, and the enzymatic mechanisms deployed by WRF during CDGs degradation have yet to be elucidated. To address this gap, the present study aimed to: (i) screen and identify potent lignocellulose-degrading fungal isolates from wild resources; (ii) characterize the genomic potential of the selected strain, *Ganoderma* sp. 2G1-6, focusing on carbohydrate-active enzymes (CAZymes); and (iii) systematically evaluate the temporal dynamics of enzymatic hydrolysis, nutritional enhancement, and microstructural deconstruction during SSF of CDGs. This research provides a theoretical basis for understanding the enzymatic machinery of WRF-driven lignocellulose degradation and offers microbial resources and strategic references for the efficient bioconversion of lignocellulosic biomass.

## 2. Materials and Methods

### 2.1. Sample Collection and Reagents

Macrofungal fruiting bodies were collected from natural environments in Chongqing, Yunnan, and Sichuan provinces, China, and stored at 4 °C prior to use.

CDGs were provided by Wuliangye Group Co., Ltd. (Yibin, China).

2,2′-Azino-bis 3-ethylbenzothiazoline-6-sulfonic acid) diammonium salt (ABTS) was purchased from Sigma-Aldrich (MO, USA). Sodium carboxymethyl cellulose (CMCNa), beechwood xylan, and 4-nitrophenyl β-D-glucopyranoside (PNPG) were obtained from Sigma-Aldrich (MO, USA), Megazyme (Bray, Ireland), and Shanghai Yuanye Bio-Technology Co., Ltd. (Shanghai, China), respectively. All chemicals were of analytical grade.

### 2.2. Isolation and Screening of Strains

Fungal strains were isolated using a tissue isolation method [18,19]. Briefly, under a laminar flow hood, collected fruiting bodies were surface-disinfected with 75% (*v*/*v*) ethanol. Each fruiting body was then aseptically torn open, and a small tissue fragment (approximately 5 mm^3^) from the cap-stem junction was excised using a sterile dissecting knife. The fragment was transferred onto a Potato Dextrose Agar (PDA) plate containing 0.1% streptomycin to inhibit bacterial growth and incubated at 30 °C. Once the mycelium fully colonized the plate, serial subculturing was performed on fresh PDA plates until pure, homogeneous, and consistently white mycelial cultures were obtained.

After cultivation on PDA, mycelial plugs were transferred to a guaiacol-based indicator medium for primary screening of laccase-producing strains with lignin-degrading potential. The indicator medium contained (*w*/*v*): 1% glucose, 0.3% malt extract, 0.3% yeast extract, 0.2% KH_2_PO_4_, 0.05% MgSO_4_·7H_2_O, 0.02% FeSO_4_·7H_2_O, 2% agar, and 0.04% guaiacol. Following incubation at 30 °C, plates were examined for a reddish-brown halo surrounding the mycelium, indicating laccase secretion.

To investigate the enzyme production characteristics of *Ganoderma* sp. 2G1-6 induced by CDGs and the synergistic relationships among the key degrading enzymes, the activities of endoglucanase, xylanase, laccase, and β-glucosidase were quantitatively measured. All assays were carried out in triplicate, and enzyme activities are reported in U/g. The selected fungal isolate was inoculated at 2% (*v*/*v*) into enzyme production medium containing (*w*/*v*): 1% CDGs, 1% wheat bran, 0.1% peptone, 0.14% (NH_4_)_2_SO_4_, 0.2% KH_2_PO_4_, 0.03% CaCl_2_, 0.03% MgSO_4_·7H_2_O, 0.0005% FeSO_4_·7H_2_O, 0.00015% MnSO_4_·7H_2_O, and 0.00015% ZnSO_4_·7H_2_O. After incubation for five days at 30 °C with shaking at 180 rpm, enzyme activity in the culture supernatant was measured.

### 2.3. Strain Identification

Following genomic DNA extraction, the 18S rDNA fragment was amplified using the primer pair NS1 (5′-GTAGTCATATGCTTGTCTC-3′) and NS6 (5′-GCATCACAGACCTGTTATTGCCTC-3′) [20], or the internal transcribed spacer (ITS) region was amplified using primers ITS1 (5′-TCCGTAGGTGAACCTGCGG-3′) and ITS4 (5′-TCCTCCGCTTATTGATATGC-3′). Sequencing was performed by Sangon Biotech (Shanghai, China). The obtained sequences were compared against the NCBI database using the online BLAST server (https://blast.ncbi.nlm.nih.gov). The 18S rDNA or ITS sequences obtained from the above-screened isolates for molecular identification have been deposited in GenBank under accession numbers PZ687935, PZ687936, PZ687948, PZ687949, PZ687950, PZ687951, PZ687958 and PZ687959.

### 2.4. Whole Genome Sequencing, Assembly, and Annotation

The genome of the fungal isolate was sequenced using Illumina short‑read and PacBio long‑read technologies. Reads were quality‑filtered with fastp (version 0.20.0), and de novo assembly was performed using SOAPdenovo2 (version 2.04) with gap filling by GapCloser (version 1.12). Gene prediction was conducted with MAKER2 (version 2.32). Functional annotation included BLAST+ (version 2.3.0) searches against the Clusters of Orthologous Genes (COG), Gene Ontology (GO), and Kyoto Encyclopedia of Genes and Genomes (KEGG) databases. Carbohydrate‑active enzymes (CAZymes) were identified using the CAZy database (version 8).

### 2.5. Solid-State Fermentation

SSF was carried out in bottles, each containing 30 g (dry matter basis) of CDGs adjusted to 70% moisture content and pH 6.0 (initial pH 3.4–3.8). The fungal strain was pre-cultured on PDA plates at 30 °C for 4–7 days. Mycelial plugs (5 mm × 5 mm) were harvested, and an inoculum equivalent to the biomass from one-eighth of a plate was added to each bottle. Fermentation was conducted at 30 °C for 5, 10, 15, 20, 25 and 30 days, with six replicate bottles prepared for each time point. At each designated time point, three replicate bottles were randomly selected for enzyme activity analysis. The contents of the remaining three replicate bottles were dried to constant weight at 65 °C and stored at 4 °C for subsequent compositional analyses (e.g., proximate analysis and lignocellulose content). An uninoculated control was processed identically at selected time points for comparison.

### 2.6. Determination of Chemical Composition

The chemical composition of raw and fermented CDGs was analyzed as follows. Cellulose content was determined according to the standard procedure of the National Renewable Energy Laboratory (NREL) [21]. Total dietary fiber content was analyzed following the method of McCleary et al. [22]. Total nitrogen content was measured using the Kjeldahl method [23], and crude protein content was calculated by multiplying the total nitrogen value by 6.25. True protein content was quantified using the copper sulfate precipitation method [24]. Dry matter loss during fermentation was determined gravimetrically.

### 2.7. Enzyme Activity Assays

Crude enzyme extracts were prepared from the solid-state fermented CDGs. Approximately 2 g of fermented material was homogenized with 20 mL of deionized water. The mixture was extracted in a shaking incubator at 30 °C and 220 rpm for 60 min. Subsequently, 2 mL of the slurry was centrifuged at 8000 rpm for 5 min, and the supernatant was collected as the crude enzyme solution for subsequent assays.

The activities of endocellulase and xylanase were determined using the 3,5-dinitrosalicylic acid (DNS) method to quantify reducing sugars [25].

β-Glucosidase activity was determined using the PNPG method according to Maués et al. [26]. One unit (U) of activity was defined as the amount of enzyme required to release 1 μmol of p-nitrophenol per minute at 40 °C and pH 5.0.

Laccase activity was assayed using ABTS as the substrate, following the method of Bao et al. [27]. One unit (U) was defined as the amount of enzyme that oxidizes 1 μmol of ABTS per minute under the assay conditions.

### 2.8. Structural Characterization

Changes in the chemical structure of CDGs before and after *Ganoderma* sp. 2G1-6 fermentation were analyzed by Fourier transform infrared spectroscopy (FTIR) using a Nicolet iN10 spectrometer (Thermo Fisher Scientific, MA, USA). Spectra were recorded in the range of 4000–400 cm^−1^ [28]. Morphological changes on the surface of CDGs were examined by scanning electron microscopy (SEM; Hitachi S-3400N, Tokyo, Japan). Samples were dried, sputter-coated with a gold layer, and observed at a magnification of 1000×. X-ray diffraction (XRD) patterns were obtained using an X’Pert Powder diffractometer (PANalytical, Almelo, The Netherlands) with a scanning range of 10–80° (2θ) at a speed of 10°/min [29]. Thermal stability was assessed by thermogravimetric analysis (TGA) using a Mettler TGA2 instrument (Mettler Toledo, Greifensee, Switzerland). Samples were heated from 30 to 800 °C at a rate of 10 °C/min under a nitrogen atmosphere [30].

### 2.9. Statistical Analysis

All enzyme activity measurements and nutritional component analyses were performed in triplicate to ensure reproducibility. Quantitative data are presented as the mean ± standard error of the mean (SEM) of at least three independent biological replicates. Data plotting was performed using Origin 27.0.0 (Origin Lab, MA, USA).

## 3. Results

### 3.1. Strain Screening and Identification

A total of eight strains were isolated and purified on PDA plates, of which six exhibited laccase activity on guaiacol indicator plates. The six laccase-positive strains were inoculated into liquid enzyme-production medium and cultured for five days. The supernatant was collected as crude enzyme solution for laccase activity assays. As shown in Table 1, strain 2G1-6 displayed the highest laccase activity (11.29 ± 0.31 U/mL) among all tested strains.

Genomic DNA was extracted from the seven strains for molecular identification. The obtained sequencing was subjected to BLAST similarity searches against the NCBI database, and the results are summarized in Table 2. Among all tested isolates, *Ganoderma* sp. 2G1-6 exhibited the highest laccase activity (11.129 U/mL). Although its endoglucanase, cellulase and xylanase activities were not the highest, its outstanding lignin-degrading potential and comprehensive lignocellulolytic enzyme profile made it the most suitable candidate. Therefore, *Ganoderma* sp. 2G1-6 was selected as the target strain for subsequent studies.

### 3.2. Genomic Sequence Characteristics

The whole genome of *Ganoderma* sp. 2G1-6 was sequenced and annotated, and an overview is presented in Table 3. The assembled genome is 49.15 Mb in size, comprising 69 scaffolds, with a GC content of 56.28%, and contains 12,437 predicted protein-coding genes.

### 3.3. Gene Function Annotation

#### 3.3.1. GO and COG Annotation

**Figure 1:**
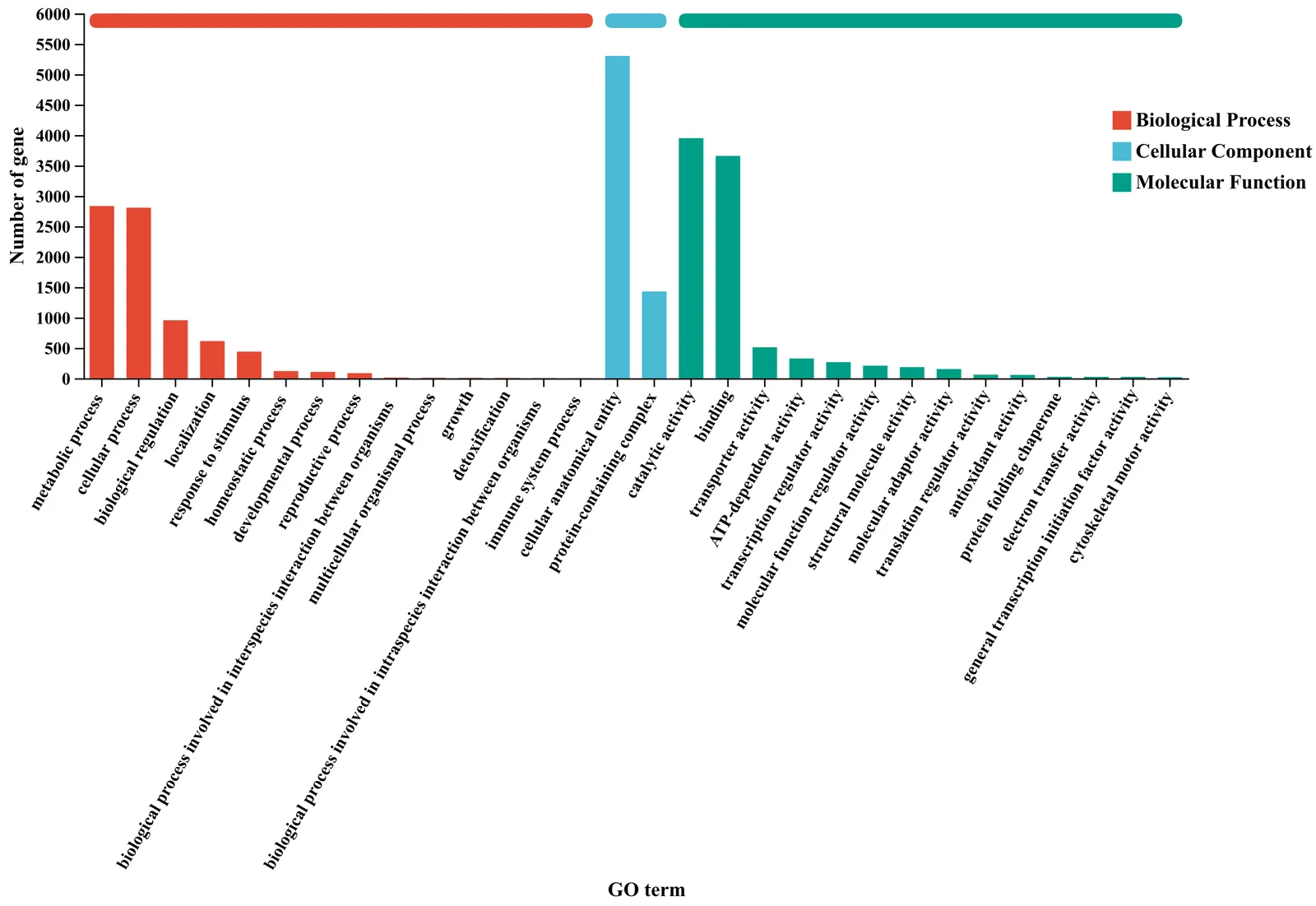
Annotation and classification of GO terms.

GO functional annotation was performed on the protein-coding genes of *Ganoderma* sp. 2G1-6. A total of 7874 genes (63.31% of all protein-coding genes) were assigned to 1929 GO terms. The annotated genes were predominantly enriched in the molecular function and biological process domains, with lower representation in the cellular component domain. Within biological processes, metabolic and cellular processes were most represented; among molecular functions, catalytic activity and binding were dominant (Figure 1).

**Figure 2:**
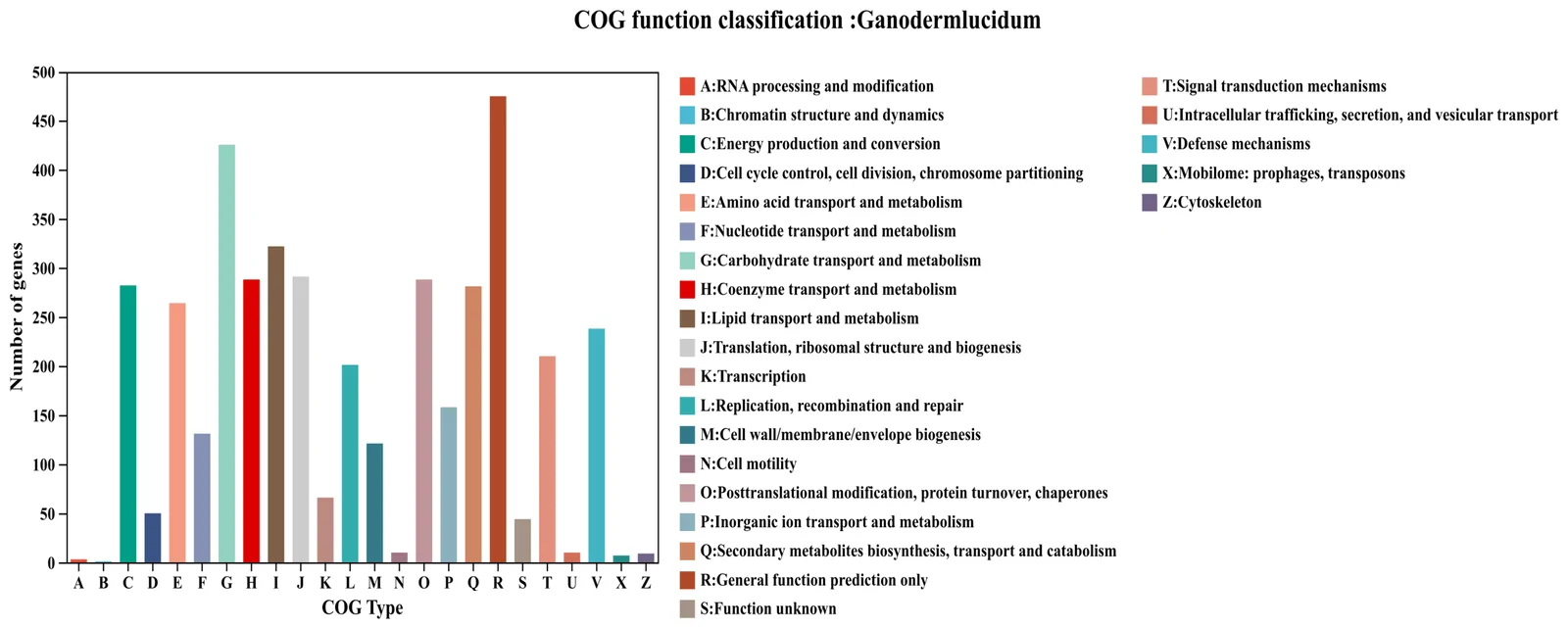
COG annotation and classification.

Genes were further annotated using the COG database. In total, 3504 genes (28.17% of all protein-coding genes) were assigned to 24 functional categories (Figure 2). The two most abundant categories were “general function prediction” (475 genes) and “carbohydrate transport and metabolism” (425 genes). The high number of genes in the latter category indicates strong carbohydrate utilization potential, which is likely central to this strain’s lignocellulose-degrading capability. Notably, the substantial proportion of genes categorized under “general function prediction” suggests that a significant portion of this functional repertoire remains uncharacterized. Genes involved in carbohydrate transport and metabolism are crucial for the breakdown and assimilation of polysaccharides and lignin-derived compounds [31].

#### 3.3.2. KEGG Annotation and CAZyme Analysis

**Figure 3:**
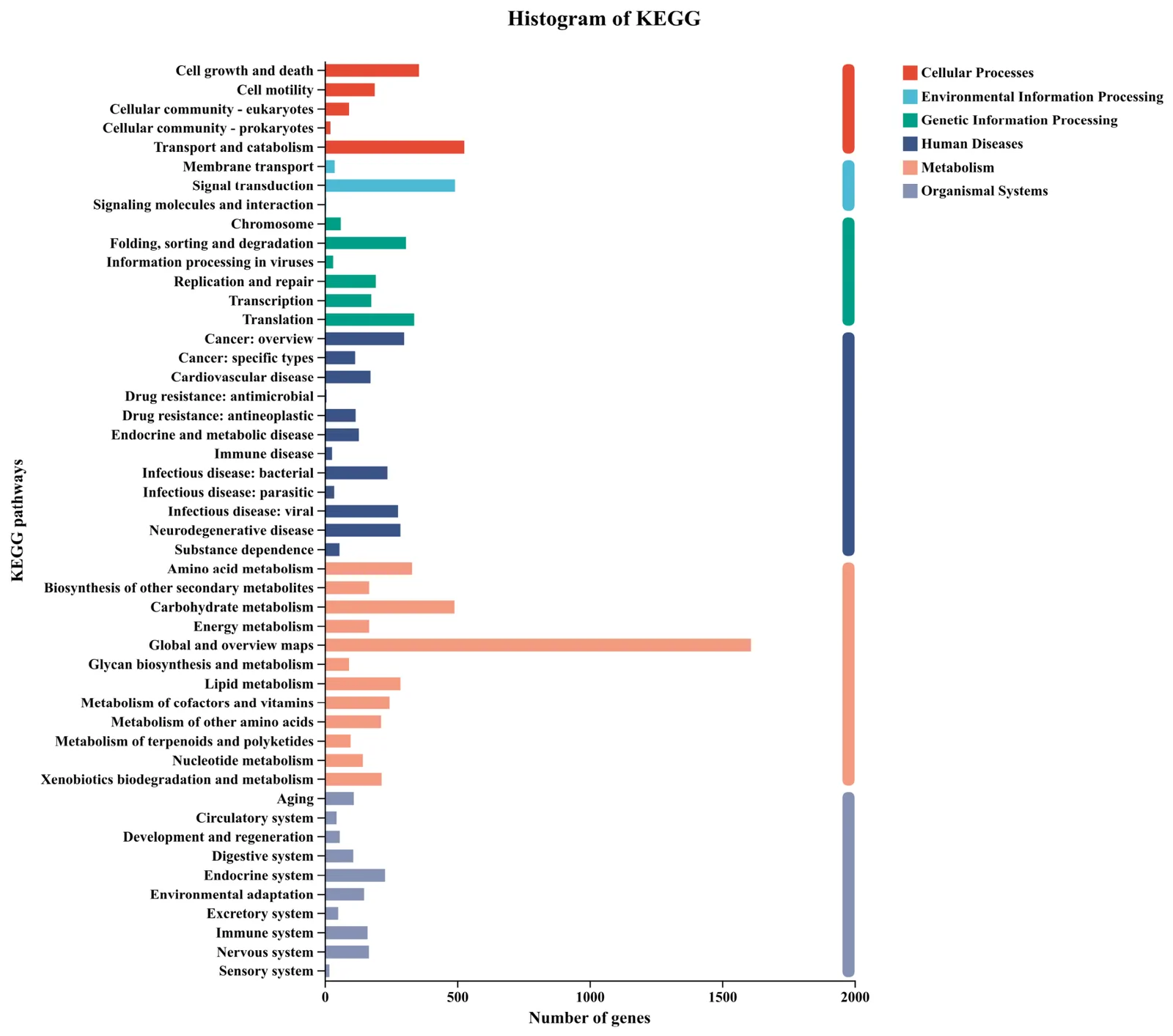
KEGG pathway functional annotation.

KEGG pathway enrichment analysis was performed to map the annotated genes. As shown in Figure 3, the genes were assigned to six major categories comprising 48 subcategories. In total, 6298 genes were mapped to 148 metabolic pathways. Among these, carbohydrate metabolism was represented by 671 genes across 16 pathways. Substantial enrichment was observed in pathways relevant to lignocellulose degradation, primarily including starch and sucrose metabolism, pyruvate metabolism, amino sugar and nucleotide sugar metabolism, propanoate metabolism, and glycolysis/gluconeogenesis.

**Figure 4:**
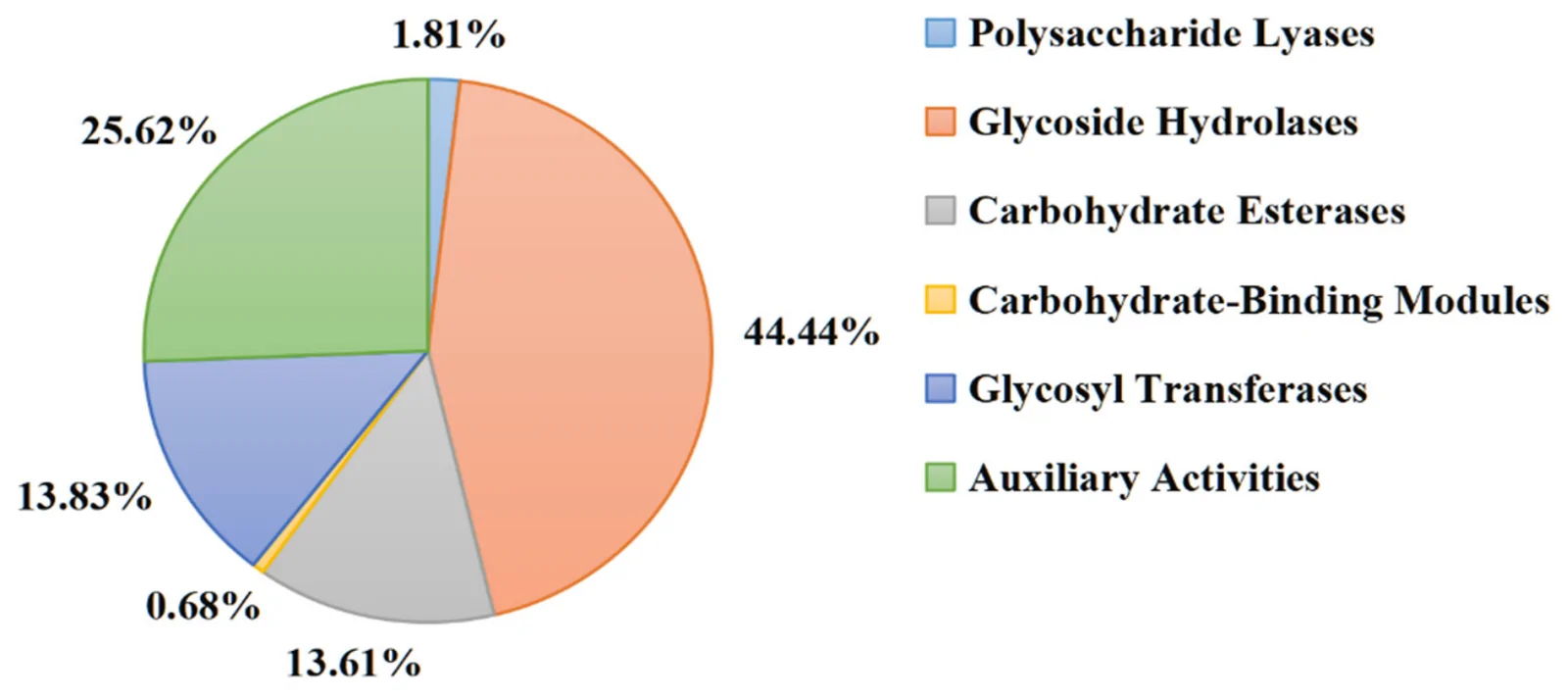
Gene count distributions of CAZy families.

CAZymes represent a central functional category in fungal genomes and are crucial for lignocellulose degradation [32]. Annotation of the *Ganoderma* sp. 2G1-6 genome identified 441 CAZymes (Figure 4), distributed among eight polysaccharide lyases (PLs), 196 glycoside hydrolases (GHs), 60 carbohydrate esterases (CEs), 61 glycosyltransferases (GTs), 113 auxiliary activities (AAs), and 3 carbohydrate-binding modules (CBMs). CBMs primarily function to anchor cellulases to cellulose surfaces, thereby facilitating cellulose degradation.

Among the CAZymes, GHs play a central role in lignocellulose degradation. Cellulases (primarily endoglucanases, cellobiohydrolases, and β-glucosidases) were represented by genes from the endoglucanase families GH5 (15 genes), GH7 (four genes), and GH6 (one gene), as well as the β-glucosidases families GH3 (three genes), GH1 (two genes), and GH9 (one gene). Hemicellulose-degrading enzymes such as xylanases were identified within families GH10 (four genes), GH55 (two genes), and GH51 (two genes), with an additional three GH3 genes implicated in xylose metabolism. Lignin degradation in white-rot fungi is primarily driven by class II heme peroxidases (AA2 family), including lignin peroxidase (LiP), manganese peroxidase (MnP), and versatile peroxidase (VP), which directly catalyze C–C and ether bond cleavage in the lignin polymer. In the genome of *Ganoderma* sp. 2G1-6, 38 genes were assigned to AA2, indicating a robust peroxidative machinery. Sixteen AA1 genes encoding laccases were also identified; laccases act on phenolic subunits and often function in concert with auxiliary oxidoreductases from families AA3, AA4, AA5, AA6, and AA9, which contribute to hydrogen peroxide production, electron transfer, and polysaccharide oxidation (Table S1). Together, these AA families form a synergistic network for complete lignocellulose deconstruction. These CAZyme ensembles are crucial for the concerted breakdown of lignocellulose [33].

In addition to lignocellulose degradation, *Ganoderma* sp. 2G1-6 are known to produce various bioactive compounds, including polysaccharides and triterpenoids [34,35,36]. Genome annotation of *Ganoderma* sp. 2G1-6 identified a repertoire of genes potentially involved in polysaccharide biosynthesis and the mevalonate (MVA) pathway for triterpenoid production, suggesting genetic capacity for these metabolites (Table S2).

### 3.4. Lignocellulolytic Enzyme Activities

**Figure 5:**
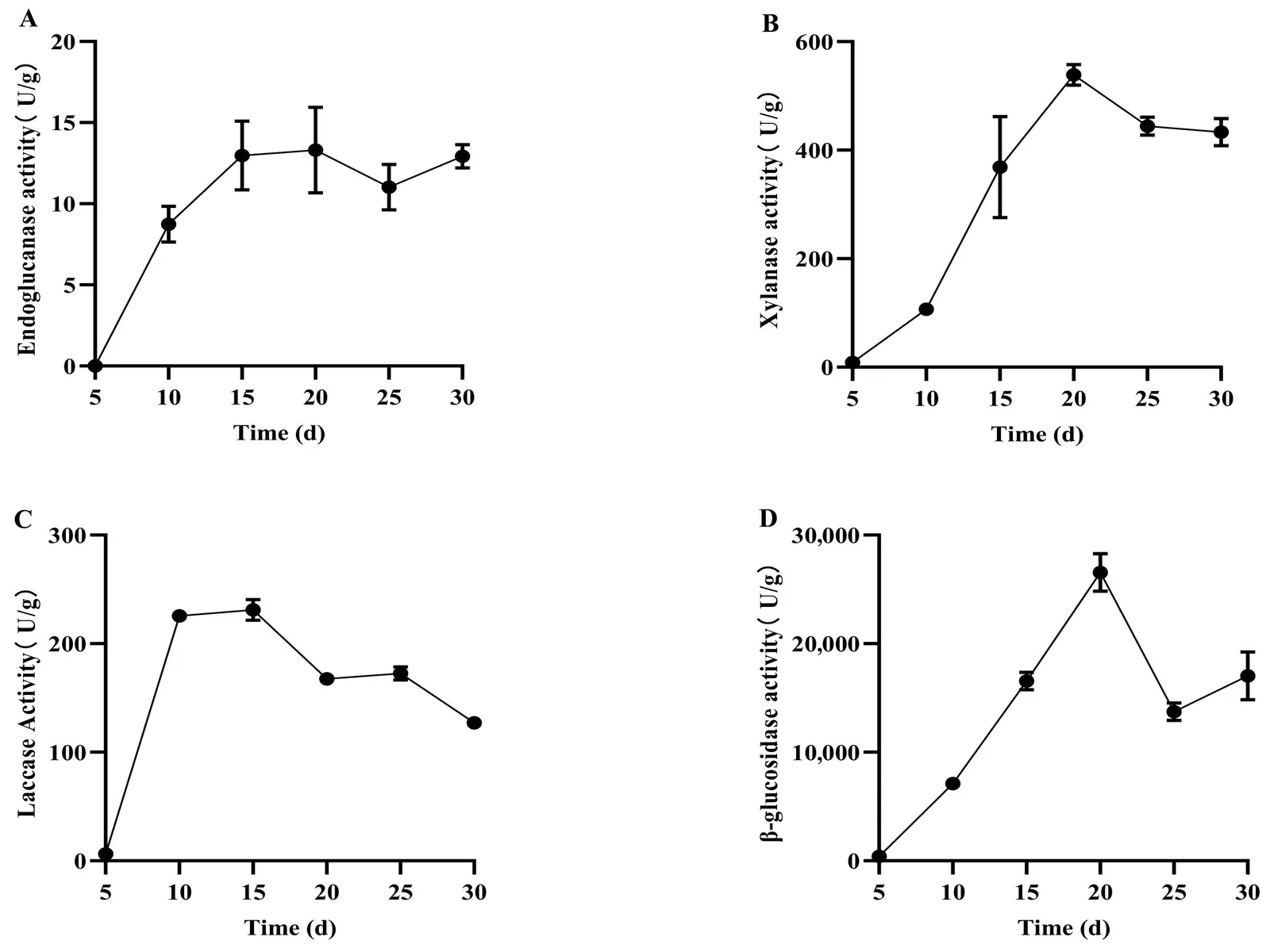
Enzyme activity trends during fermentation of *Ganoderma* sp. 2G1-6 in Chinese distillers’ grains. (**A**) Endoglucanase activities; (**B**) xylanase activities; (**C**) laccase activities; (**D**) β-glucosidase activities.

During the SSF of CDGs by *Ganoderma* sp. 2G1-6, the activities of key lignocellulose-degrading enzymes changed dynamically (Figure 5). Endoglucanase activity, which hydrolyzes β-1,4-glycosidic bonds in cellulose, exhibited a fluctuating trend and reached a maximum of 13.3 ± 4.6 U/g on day 20, followed by a gradual decline and stabilization (Figure 5A). Xylanase activity, targeting hemicellulosic xylan, peaked on day 20 at 538.8 ± 33.0 U/g, followed by a gradual decline and stabilization (Figure 5B). β-Glucosidase activity, responsible for converting oligosaccharides to glucose, showed a similar pattern, reaching a maximum of 26,572 ± 2996 U/g on day 20 (Figure 5D). In contrast, laccase activity displayed a distinct temporal profile, peaking earlier at 231.2 ± 16.7 U/g on day 15 (Figure 5C).

### 3.5. Fermentation-Induced Changes in Chemical Composition

**Figure 6:**
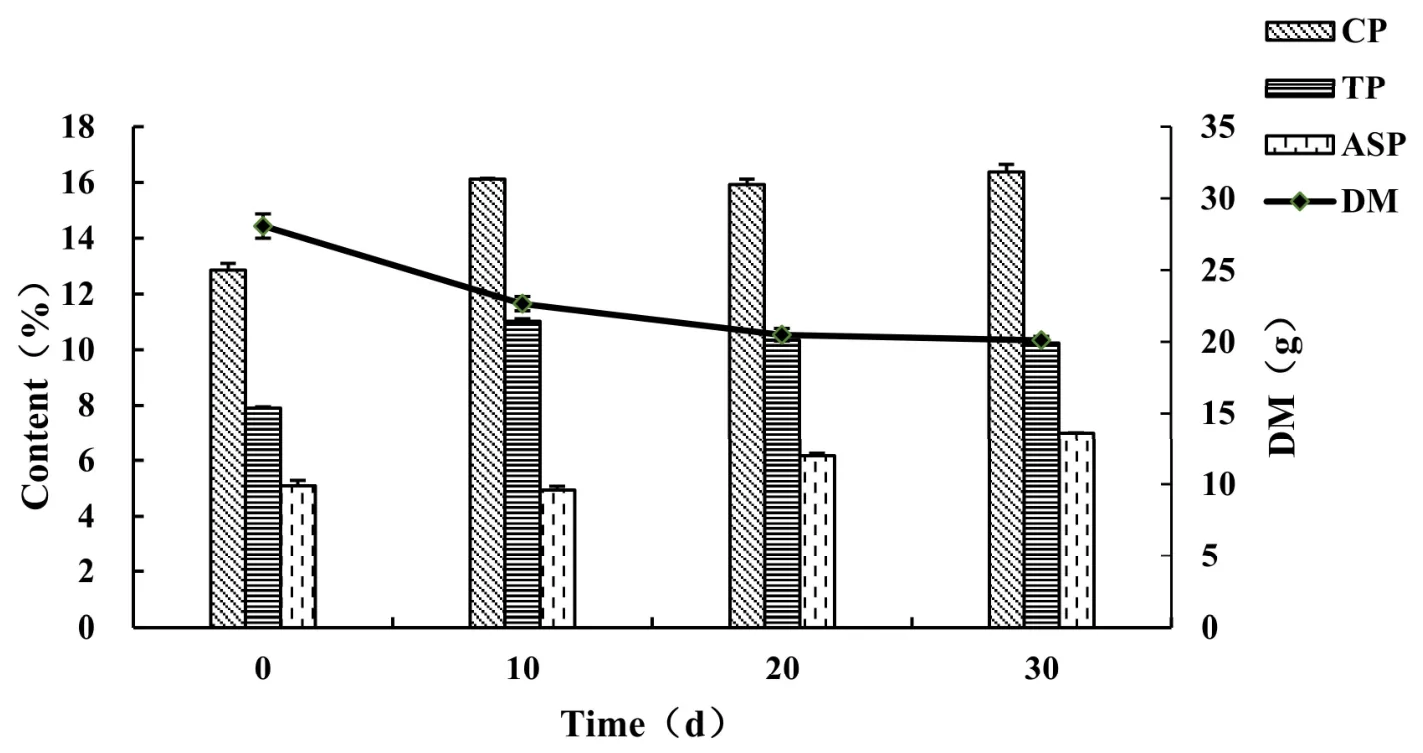
Trends in dry matter and protein content during fermentation of *Ganoderma* sp. 2G1-6 in Chinese distillers’ grains.

**Figure 7:**
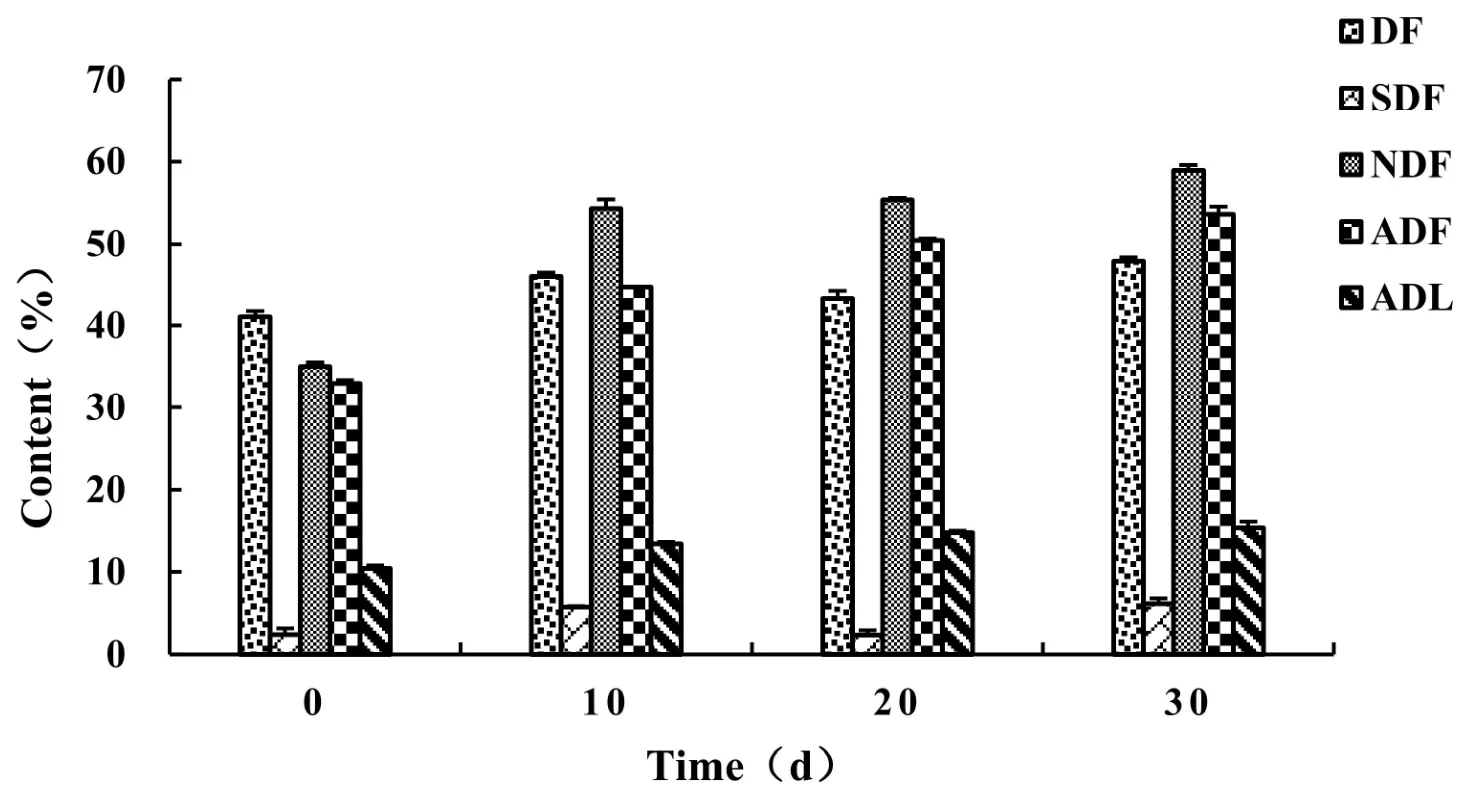
Trends in lignocellulose changes during the fermentation of *Ganoderma* sp. 2G1-6 in Chinese distillers’ grains. Dietary fiber (DF), soluble dietary fiber (SDF), neutral detergent fiber (NDF), acid detergent fiber (ADF), acid detergent lignin (ADL).

Changes in the nutritional composition of CDGs during SSF with *Ganoderma* sp. were analyzed. Fermentation significantly increased the content of all measured protein components (Figure 6). Crude protein (CP) and acid-soluble protein (ASP) contents increased progressively with fermentation time, reaching gains of 28.51% and 33.33%, respectively, by day 30. In contrast, true protein (TP; the protein fraction after removing non-protein nitrogen) content peaked earlier at day 10, with a 39.32% increase. Concurrently, dry matter (DM) loss gradually increased over time. Despite this loss, lignocellulose-associated components showed substantial modification (Figure 7), most notably a 159% increase in soluble dietary fiber (SDF) content after 30 days of fermentation.

### 3.6. Spectroscopic Analysis

**Figure 8:**
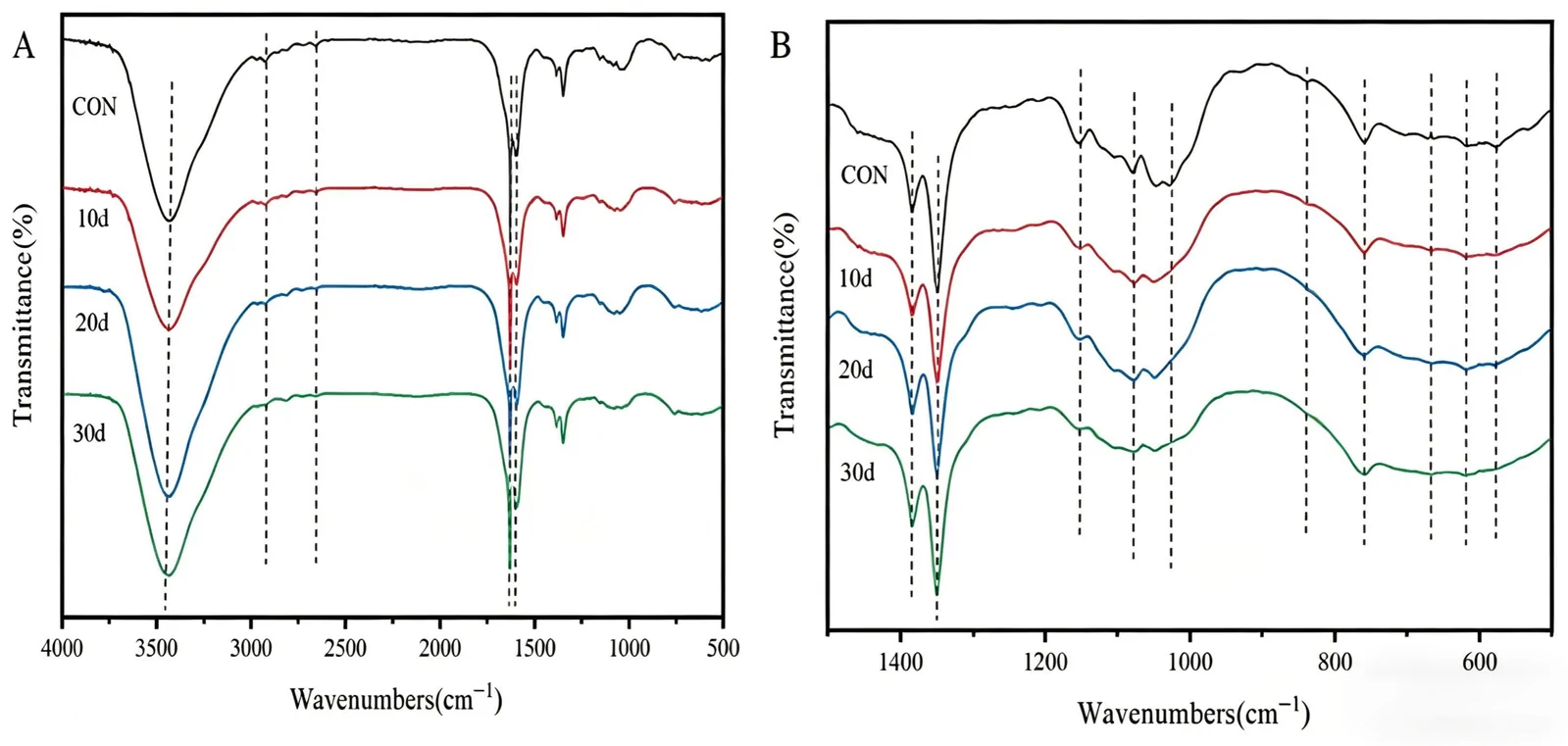
FTIR spectra of fermented Chinese distillers’ grains (CDGs) from *Ganoderma* sp. 2G1-6 (**A**) FTIR spectrum of CDGs at 500–4000 cm^−1^; (**B**) FTIR spectrum of CDGs at 500–1500 cm^−1^.

FTIR spectroscopy was employed to monitor chemical structural changes in CDGs during *Ganoderma* sp. 2G1-6 fermentation (Figure 8) [37]. The spectra displayed characteristic lignocellulose bands, with cellulose and hemicellulose contributing O-H stretching at 3434 cm^−1^ and C-H stretching at 2928 cm^−1^ (Figure 8A), and lignin contributing aromatic C=C stretching at 1632 and 1599 cm^−1^ (Figure 8B), as well as guaiacyl ring vibrations at 1349 cm^−1^ [38,39]. Spectral variations indicated progressive degradation of cellulose and hemicellulose: the intensity of the 3434 cm^−1^ band increased over time, suggesting greater exposure of cellulose hydroxyl groups [38], while polysaccharide-associated bands at 2928 cm^−1^ (C-H in CH_3_/CH_2_), 1079 cm^−1^ (asymmetric C-O-C stretching), 1024 cm^−1^ (C-O stretching in primary alcohols), and low-frequency peaks at 759 cm^−1^ and 575 cm^−1^ (β-1,4-glycosidic bonds) gradually weakened or disappeared [39,40,41,42].

Concurrently, lignin structural modifications were evidenced by the reduced intensity of bands at 2928 cm^−1^ (attributed to lignin side-chains) and 1024 cm^−1^ (associated with guaiacol C-H stretching), indicating degradation of methyl/methylene groups and guaiacol structures [41,43]. The attenuation of the 1384 cm^−1^ (C-H stretching) and 1349 cm^−1^ peaks further corroborated alteration of the lignin structure.

### 3.7. Scanning Electron Microscopy

**Figure 9:**
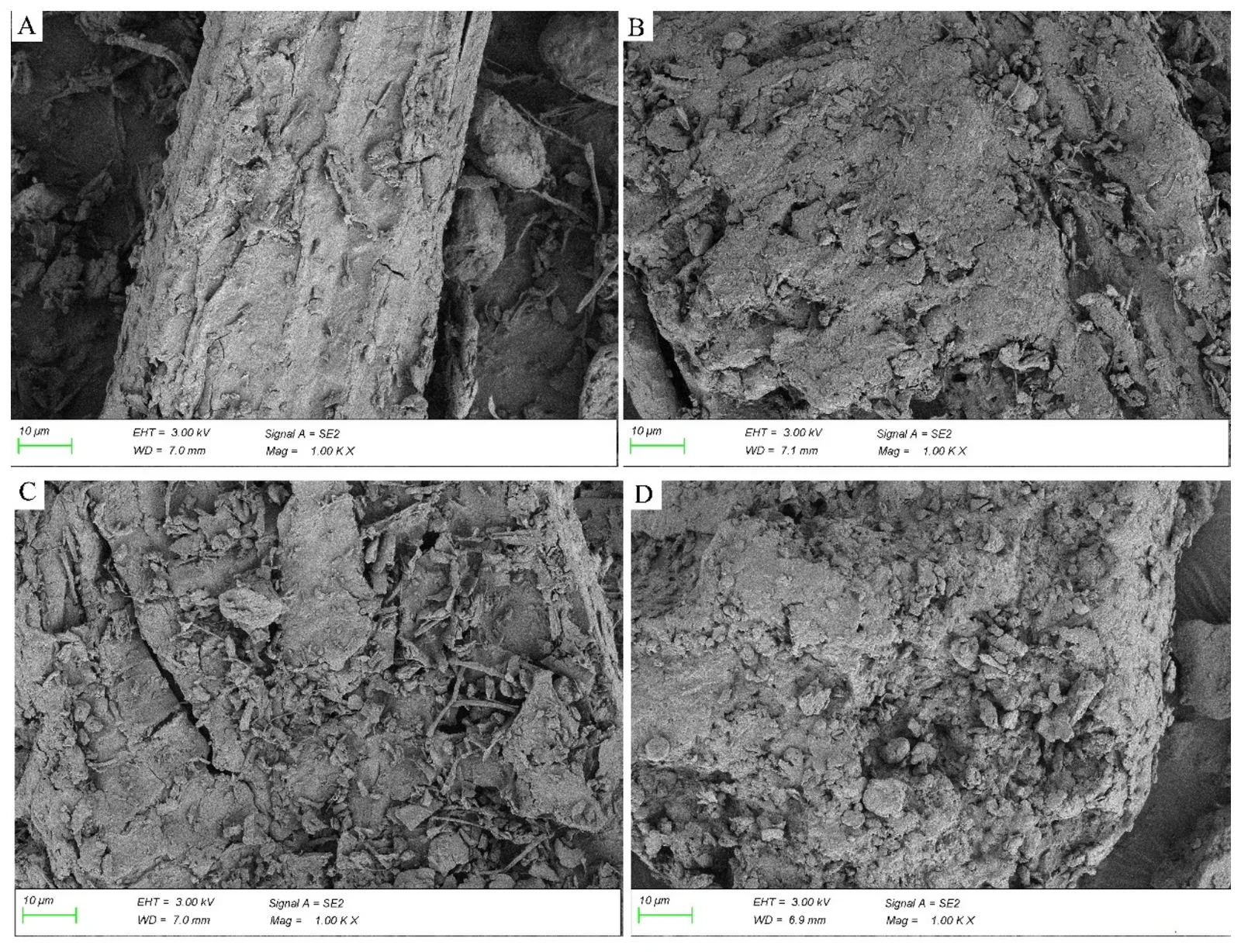
SEM Images of Chinese distillers’ grains (CDGs) from *Ganoderma* sp 2G1-6 after fermentation. (**A**) SEM micrograph of CDGs before fermentation; (**B**) SEM micrograph of CDGs after 10 days of fermentation; (**C**) SEM micrograph of CDGs after 20 days of fermentation; (**D**) SEM micrograph of CDGs after 30 days of fermentation.

SEM revealed significant alterations in the surface microstructure of CDGs following fermentation with *Ganoderma* sp. (Figure 9). Untreated CDGs exhibited an intact, dense, and non-porous surface (Figure 9A), in which cellulose appeared to be embedded within a cohesive matrix of lignin, hemicellulose, and other components, thereby limiting cellulase accessibility. In contrast, after fermentation, the surface morphology was markedly transformed, becoming rough and uneven with the appearance of numerous pores and structural fragments of varying sizes (Figure 9B–D). This structural disruption exposed internal fiber bundles, thereby significantly enhancing the accessibility of cellulase to its substrate.

### 3.8. X-Ray Diffraction Analysis

**Figure 10:**
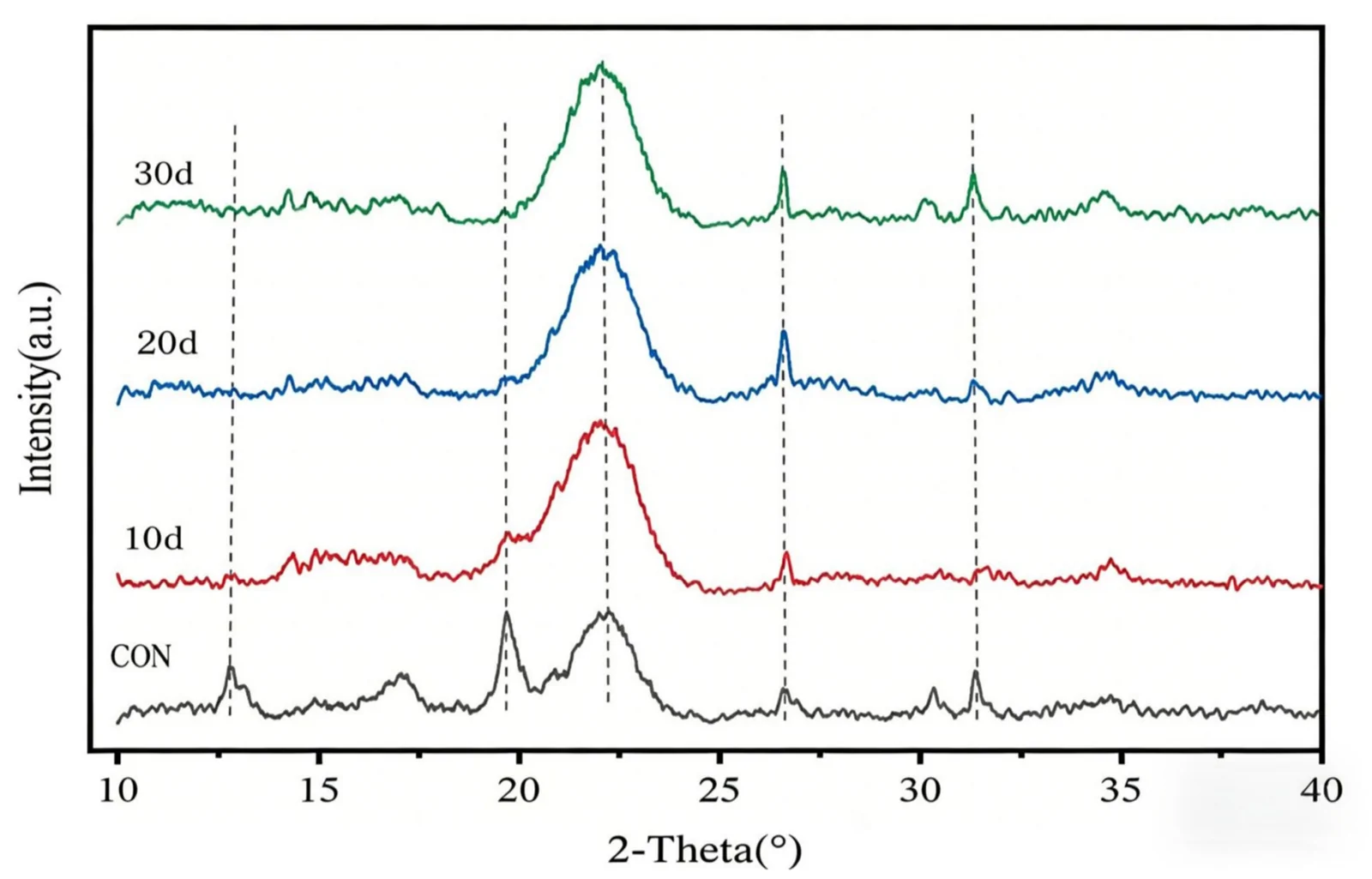
XRD pattern of fermented Chinese distillers’ grains from *Ganoderma* sp.

The crystalline structure of CDGs before and after fermentation was analyzed by XRD (Figure 10). Raw CDGs exhibited characteristic diffraction peaks at 2θ = 12.7°, 19°, 22°, 26°, and 31°. With increasing fermentation time, distinct changes were observed. The peaks at 12.7° and 19°, associated with amorphous components [44], progressively weakened and eventually disappeared. The peak at 31° showed a similar decreasing trend.

In contrast, the diffraction peaks at 22° and 26°, indicative of crystalline cellulose regions, progressively intensified. The enhancement of the peak near 22° suggests an increase in relative cellulose crystallinity. This is consistent with selective degradation of amorphous components (e.g., non-cellulosic polysaccharides and lignin) by *Ganoderma* sp., which enriches the relative proportion of crystalline cellulose in the residual material. This finding aligns with the structural compaction effect reported by Wang et al. [45].

### 3.9. Thermogravimetric Analysis

**Figure 11:**
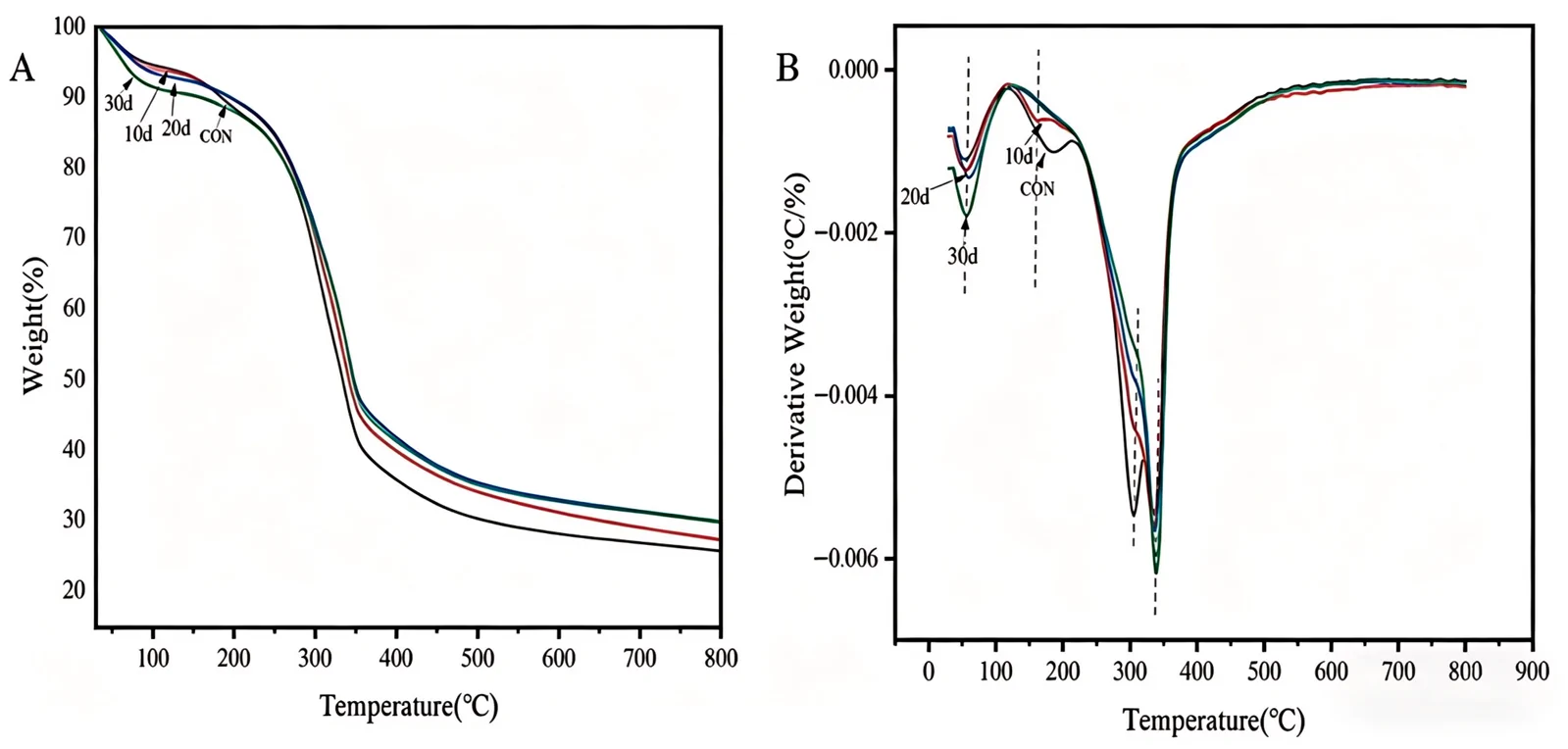
Thermogravimetric analysis results of *Ganoderma* sp. 2G1-6 fermented Chinese distillers’ grains (CDGs). (**A**) TGA curve of *Ganoderma* sp. 2G1-6 fermented CDGs; (**B**) DTG analysis curve of *Ganoderma* sp. 2G1-6 fermented CDGs.

TGA and derivative thermogravimetric (DTG) analyses were used to characterize the pyrolysis profiles of CDGs at different fermentation stages (Figure 11). Weight loss occurred in three main stages: initial moisture evaporation (<150 °C), primary devolatilization of lignocellulose (150–500 °C), and subsequent carbonization of residues (>400 °C) (Figure 11A).

With extended fermentation time, the rate of weight loss in the first stage increased, consistent with higher moisture content in the fermented biomass. The most pronounced changes were observed in the primary devolatilization stage (150–500 °C), where the majority of mass loss occurred (Figure 11A). DTG curves provided further resolution: unfermented CDGs (control) exhibited a minor shoulder peak at 150–250 °C, attributable to hemicellulose decomposition, and two distinct peaks near 300 °C and 350 °C, corresponding to the degradation of cellulose, lignin, and starch (Figure 11B).

Fermentation progressively eliminated the low-temperature shoulder peak (150–250 °C), indicating substantial hemicellulose degradation [46]. Concurrently, the shoulder peak near 300 °C diminished, suggesting removal of amorphous hemicellulose, lignin, and some non-crystalline cellulose. In contrast, the DTG peak near 350 °C, associated with crystalline cellulose, intensified with fermentation time. This shift in thermal degradation profiles indicates that *Ganoderma* sp. fermentation preferentially removed amorphous components, thereby increasing the relative proportion and thermal stability of crystalline cellulose in the residual substrate [47].

## 4. Discussion

The effective degradation of lignocellulose, a major component of CDGs, is crucial for their resource utilization. Although both fungi and bacteria exhibit inherent lignocellulose-degrading capabilities, their degradation mechanisms diverge considerably. Most aerobic bacteria secrete limited enzyme sets, often with low catalytic efficiency and practical purification difficulties. [48]. Anaerobic bacteria, although resistant to contamination, typically exhibit slow growth [49]. In contrast, fungi, particularly WRF, produce a potent and synergistic suite of extracellular lignocellulolytic enzymes, making them more effective than genera such as *Trichoderma* or *Aspergillus* in decomposing natural substrates [50]. The structural complexity of lignocellulose necessitates such multi-enzyme systems for complete hydrolysis [51].

In this study, a WRF strain (*Ganoderma* sp. 2G1-6) was isolated and its genome sequenced, revealing genetic potential for lignocellulose degradation. This potential was experimentally verified by monitoring dynamic changes in key enzyme activities and nutritional components during the SSF of CDGs. In addition, multi-faceted physicochemical characterization provided microstructural evidence of substrate degradation. Collectively, these approaches characterized the enzymatic and structural dynamics of lignocellulose degradation by this WRF in CDGs.

Whole-genome sequencing of *Ganoderma* sp. 2G1-6 provided comprehensive insights into its lignocellulolytic potential. The assembled genome size of 49.15 Mb falls within the typical range reported for *Ganoderma* sp. (30–50 Mb) [51,52]. Functional annotation revealed a substantial genetic repertoire, including 7874 GO terms, 3504 COG (KOG) entries, and 6425 KEGG pathways, comparable to other sequenced WRF such as *Irpex lacteus* J2 [53]. Notably, the annotation highlighted a pronounced predisposition toward carbohydrate metabolism.

A key finding was the identification of 441 CAZymes, exceeding the numbers reported for some well-studied WRF such as *Hypoxylon flavopileum* (over 400 CAZymes) [54]. These CAZymes span at least 119 families and include a robust set of 196 GHs critical for cellulose and hemicellulose deconstruction. This GH repertoire comprises endoglucanases, exoglucanases, and β-glucosidases, which act synergistically to hydrolyze cellulose chains to glucose [55,56].

Equally important for lignocellulose breakdown is the lignin-degrading apparatus. The genome encodes 113 AA enzymes, placing this strain in the upper range reported for WRF (70–115) and substantially exceeding typical numbers in brown rot or soft rot fungi [57]. Among these, the class II peroxidases (38 AA2 genes), including lignin peroxidases and manganese peroxidases, are considered the principal lignin depolymerizers capable of cleaving C–C and ether bonds. Sixteen AA1 laccase genes were also identified, together with 33 AA3 family genes (e.g., GMC oxidoreductases, alcohol oxidases) involved in hydrogen peroxide supply, and 12 AA9 lytic polysaccharide monooxygenases that catalyze oxidative cleavage of polysaccharides [58]. In summary, the genomic landscape of *Ganoderma* sp. 2G1-6 depicts a robust and balanced enzymatic toolkit for lignocellulose degradation, featuring a rich diversity of CAZymes for polysaccharide hydrolysis alongside a comprehensive oxidative enzyme network that extends beyond individual enzyme families.

The degradation of lignocellulose in CDGs requires the synergistic action of multiple enzymes secreted by *Ganoderma* sp. 2G1-6. Monitoring this process revealed dynamic changes in enzyme activities. After an initial period of low activity, endoglucanase, xylanase, and β-glucosidase activities all increased, peaking around day 20 (13.3 ± 4.6 U/g, 538.8 ± 33.0 U/g, and 26,572 ± 2996 U/g, respectively) before gradually declining. This increase-peak-decline pattern is consistent with reports for other fungi, such as *Pleurotus ostreatus* [59,60], although the peak activities observed here, particularly for endoglucanase, were higher than some previous reports [59]. Notably, xylanase activity in this study also exceeded levels reported for *Irpex lacteus* and *Phlebia acerina* on other substrates [61].

The temporal pattern of laccase activity observed in the present study parallels that documented for other WRF, including *Pleurotus florida* and *Ganoderma* sp. [62], peaking at day 15 (231.2 ± 16.7 U/g) and then decreasing. The exceptionally high β-glucosidase activity is notable, as it facilitates hydrolysis of inhibitory disaccharides and, thereby, promotes more efficient cellulose breakdown [63]. In the plant cell wall, lignin fills the cellulose framework and is covalently bound to hemicellulose, forming a barrier to hinder cellulose degradation. The early oxidative modification of lignin phenolic units by laccase may partially destabilize the phenolic hydroxyl structures, thus improving the accessibility of cellulases and hemicellulases to lignocellulosic substrates and enhancing cellulase catalytic efficiency. Notably, the cleavage of C–C bonds in the lignin backbone depends primarily on peroxidases (e.g., lignin peroxidase and manganese peroxidase), whereas laccase functions as a lignin-modifying enzyme that prepares the substrate for subsequent enzymatic degradation. Alternatively, lignin-degrading enzymes may initially suppress cellulase expression, with cellulase activity rising only after laccase declines, as reported in other studies [64,65,66]. Laccase, xylanase, endoglucanase and β-glucosidase showed a clear order of action in the degradation of lignocellulose in CDGs. Laccase preferentially acts on the lignin network and removes its hindrance; xylanase then degrades the main chain of hemicellulose and exposes the cellulose skeleton; the endoglucanase further cuts the amorphous region of cellulose and increases the accessible sites; the β-glucosidase completes the final conversion of cellobioses to glucose [67]. The SEM results showed that the surface structure of the CDGs was obviously broken and the porosity was significantly increased after the action of laccase and cellulase, which confirmed the synergistic effect of the two in the process of lignocellulose degradation. In thermogravimetric analysis, the degradation of hemicellulose components was found, which may be the result of the action of xylanase and β-glucosidase. High xylanase, β-glucosidase and laccase activities reduced the content of hemicellulose and lignin components in CDGs. The activity of endoglucanase was relatively low, and the degradation ability of cellulose was weak, which was consistent with the results of FTIR analysis and XRD analysis.

Further analysis focused on revealed compositional changes in CDGs during fermentation. The contents of CP, TP and ASP all increased in the fermented material, consistent with reports that WRF treatment elevates the CP content in wheat straw and rice straw [68,69]. This increase is likely due to utilization of substrate nitrogen by *Ganoderma* sp. 2G1-6 for protein synthesis to support mycelial proliferation. Concurrently, SDF content increased progressively. This conversion is consistent with SSF of other agro-residues by *Ganoderma* sp. [70] and can be attributed to the action of cellulase, β-glucosidase, and laccase secreted during fermentation. These enzymes degrade cellulose and hemicellulose, thereby promoting the release or formation of soluble fiber components [71].

Interestingly, despite enzymatic degradation, the measured contents of NDF, ADF, and ADL exhibited varying degrees of increase. This apparent contradiction can be explained by the dynamics of SSF. Although *Ganoderma* sp. 2G1-6 degrades lignocellulose components, as demonstrated by substantial lignin, cellulose, and hemicellulose degradation in other substrates [72], overall DM is simultaneously lost through fungal metabolism. If the rate of total DM loss exceeds the absolute degradation rate of the lignocellulosic fractions, their relative proportions in the residual substrate can increase [73].

The combination of microscopic techniques (FTIR, SEM, XRD, and TGA) was employed to elucidate the structural transformation and degradation mechanism of CDGs during *Ganoderma* sp. 2G1-6 fermentation. FTIR and XRD analyses revealed disruption of the lignocellulosic matrix and increased exposure of cellulose. This structural alteration was directly corroborated by SEM, which showed transformation of the initial compact, non-porous surface into a rough, porous morphology with exposed fiber bundles, thereby significantly enhancing cellulose accessibility. The observed changes are attributed to preferential degradation of amorphous components.

TGA supported this interpretation, indicating reduced pyrolytic mass loss from hemicellulose and a relative increase from cellulose, consistent with selective removal of amorphous lignin and hemicellulose. This selective degradation also explains the apparent increase in cellulose crystallinity observed by XRD, a phenomenon reported for other fungi [64]. One contributing factor may be the relatively high secretion of xylanase, β-glucosidase, and laccase compared with that of cellulase, favoring degradation of the amorphous matrix over crystalline cellulose. The resulting porous structure, with increased specific surface area, likely improves water and oil retention capacity [73], which may correlate with the elevated initial weight loss rate observed in the TGA. Collectively, these analyses demonstrate that *Ganoderma* sp. 2G1-6 enhances CDG degradability by selectively dismantling the amorphous lignin-hemicellulose matrix, disrupting its compact architecture, and thereby improving cellulose accessibility and relative crystallinity [73].

## 5. Conclusions

Herein, *Ganoderma* sp. 2G1-6 was isolated from a natural environment. Whole-genome sequencing revealed that this strain exhibits robust lignocellulose-degrading potential and possesses genetic capacity for bioactive compound synthesis. As a proof of concept, *Ganoderma* sp. 2G1-6 was co-cultured with CDGs in SSF. The results demonstrated high activities of endoglucanase, xylanase, β-glucosidase, and laccase. Concurrently, protein components increased, while progressive degradation of fibrous structures was observed. With prolonged fermentation, the compact substrate architecture was disrupted, and amorphous cellulose was degraded alongside lignin. Collectively, these results establish *Ganoderma* sp. 2G1-6 as an efficient lignocellulose degrader and a promising fungal chassis for the bioconversion of recalcitrant agricultural residues. This work expands the known enzymatic repertoire of *Ganoderma* sp. and provides a foundation for future studies on the functional genomics and biotechnological application of white-rot fungi in lignocellulose valorization.

## Figures and Tables

**Table 1:** Fermentation and enzyme production of the strains.

Strain Number	Endoglucanase Activities (U/mL)	Xylanase Activities (U/mL)	Laccase Activity (U/mL)
1G2-2	0.38 ± 0.09	0.23 ± 0.04	0.53 ± 0.02
2G1-6	0.88 ± 0.03	2.8 ± 0.18	11.29 ± 0.31
5G8	1.83 ± 0.1	4.48 ± 0.24	3.52 ± 0.16
5G3-2	0.38 ± 0.03	2.22 ± 0.67	0.05 ± 0
5G11	2.62 ± 0.06	47.82 ± 0.88	0.29 ± 0.01
2G2-1	0.99 ± 0.01	6.21 ± 0.06	0
5G14	2.48 ± 0.03	62.05 ± 1.82	0
CT1	2.94 ± 0.15	4.97 ± 0.18	0.94 ± 0.05

**Table 2:** BLAST alignment results for strain identification.

NO.	Strain Number	Scientific Name	GenBank Accession No.
1	1G2-2	*Perenniporia tephropora*	PZ687948
2	2G1-6	*Ganoderma* sp.	PZ687935
3	5G8	*Trametes sanguinea*	PZ687949
4	5G3-2	*Microporus xanthopus*	PZ687936
5	5G11	*Trichoderma harzianum*	PZ687958
6	2G2-1	*Phanerochaete sordida*	PZ687950
7	5G14	*Trichoderma citrinoviride*	PZ687951
8	CT1	*Coriolopsis trogii*	PZ687959

**Table 3:** Genomic features of *Ganoderma* sp. 2G1-6.

Features	Value
Genome size(bp)	49,155,725
Gene internal GC content(%)	56.28
Scaffold number	69
Gene number	12,437
GO annotation	7874
KEGG annotation	6425
COGs annotation	3504
CAZy annotation	441
P450 annotation	349
tRNA	343
5S rRNA	23
5.8S rRNA	21
18S rRNA	20
28S rRNA	22

## Data Availability

The data presented in the manuscript are available on request from the corresponding author.

## Supplementary Materials

The following supporting information can be downloaded at https://www.mdpi.com/article/10.3390/jof12080574/s1: Table S1: Annotated genes in the lignocellulose degradation pathway of *Ganoderma* sp. 2G1-6.; Table S2: Genes involved in polysaccharide synthesis in *Ganoderma* sp. 2G1-6.
