# Supplementary material for: Screening White-Rot Fungi and Characterizing Lignocellulose Degradation by Ganoderma sp. from Chinese Distillers’ Grains
Source: J Fungi (Basel). 2026 Aug 3;12(8):574. doi: 10.3390/jof12080574 (PMC13514402; doi:10.3390/jof12080574)
Supplement: Supplementary file 1 [file jof-12-00574-s001.zip › jof-4421956-supplementary.pdf]

**Table S1.** Annotated genes in the lignocellulose degradation pathway of *Ganoderma* sp. 2G1-6

| Classification | Function description | CAZy family | Enzyme Commission number | Total |
|---|---|---|---|---|
| Cellulose-related | endo-beta-1,4-glucanase | GH131,GH5,GH7,GH9 | EC 3.2.1.4 | 8 |
| | cellobiohydrolase<br>reducing end-acting cellobiohydrolase | GH6,GH7,GH9 | EC 3.2.1.91<br>EC 3.2.1.176 | 6 |
| | beta-glucosidase | GH3,GH5,GH1 | EC 3.2.1.21 | 7 |
| Hemicellulose-related | endo-1,4-beta-xylanase | GH10 | EC 3.2.1.8 | 4 |
| | beta-xylosidase<br>xylan 1,4-beta-xylosidase | GH3,GH51,GH1 | EC 3.2.1.37<br>EC 3.2.1.37 | 7 |
| | xylan alpha-1,2-glucuronidase | GH151 | EC 3.2.1.131 | 2 |
| | alpha-L-arabinofuranosidase | GH3,GH51,GH2 | EC 3.2.1.55 | 9 |
| | acetyl xylan esterase | CE4,CE1,CE12 | EC 3.1.1.72 | 12 |
| | mannan endo-1,4-beta-mannosidase | GH5 | EC 3.2.1.78 | 1 |
| | beta-mannosidase | GH1,GH5,GH2 | EC 3.2.1.25 | 7 |
| Lignin-related | Laccase | AA1 | EC 1.10.3.2 | 16 |
| | lignin peroxidase | AA2 | EC 1.11.1.14 | 19 |
| | manganese peroxidase | AA2 | EC 1.11.1.13 | 19 |
| | alcohol oxidase | AA3,AA5 | EC 1.1.3.13 | 15 |
| | alcohol oxidase-like protein | AA3 | - | 2 |
| | GMC oxidoreductase | AA3 | - | 17 |
| | choline dehydrogenase | AA3 | EC:1.1.99.1 | 1 |
| | pyranose 2-oxidase | AA3 | EC 1.1.3.10 | 1 |
| | vanillyl-alcohol oxidase | AA4 | EC 1.1.3.38 | 2 |
| | glyoxal oxidase | AA5 | EC 1.2.3.15 | 5 |
| | 1,4-benzoquinone reductase | AA6 | EC. 1.6.5.6 | 2 |
| | lytic polysaccharide monooxygenases | AA9 | EC 1.14.99.54 | 12 |

**Table S2.** Genes involved in polysaccharide synthesis in *Ganoderma* sp. 2G1-6

| Gene Name | Number | Functions |
|---|---|---|
| phosphoglucomutase | 2 | Catalyzing the Synthesis of UDP-Glucose |
| UDPglucose--hexose-1-phosphate uridylyltransferase | 1 | |
| UTP--glucose-1-phosphate uridylyltransferase | 3 | |
| phosphomannomutase | 2 | |

| | | |
|---|---|---|
| mannose-1-phosphate guanylyltransferase | 1 | Catalyzing GDP-mannose synthesis |
| UDP-sugar pyrophosphorylase | 4 | Synthesize various UDP-sugars using UTP and glucose-1-phosphate |
| glycogen synthase | 1 | Catalyzing the elongation of glycogen chains |
| alpha-1,2-mannosyltransferase | 3 | Catalytic extension of mannose chains |
| glycogenin | 7 | Initiate glycogen molecule formation |
| beta-1,4-mannosyltransferase | 1 | Catalytic β-linked Mannan Synthesis |
